# Starvation in obsessive–compulsive disorder due to scrupulosity

**DOI:** 10.4103/0019-5545.31563

**Published:** 2006

**Authors:** Dinesh Dutt Sharma, Ramesh Kumar, Ravi Chand Sharma

**Affiliations:** * Ex-Senior Resident, Department of Psychiatry, Indira Gandhi Medical College, Shimla, Himachal Pradesh; ** Assistant Professor, Department of Psychiatry, Indira Gandhi Medical College, Shimla, Himachal Pradesh; *** Professor and Head, Department of Psychiatry, Indira Gandhi Medical College, Shimla, Himachal Pradesh

**Keywords:** Starvation, obsessive–compulsive disorder, scrupulosity

## Abstract

This report describes an unusual presentation of obsessive–compulsive disorder (OCD) with predominant religious obsessions and compulsions (scrupulosity) in which the patient starved himself by keeping fast excessively to the extent of emaciation and extreme weakness even in walking and became bedridden.

## INTRODUCTION

Obsessive–compulsive disorder (OCD), previously considered relatively a rare disorder, is quite common and has a lifetime prevalence rate of 2.5%.^1^ There are different types of obsessions and compulsions. Religious obsessions and compulsions (scrupulosity) lead to rigid ritualized behaviour where religious beliefs become compulsive and joyless and the person is unable to feel forgiven for un-confessed sins.^2^ Although slowness results from most of the rituals and is a common disability in this disorder, religious ritualized behavior leading to starvation has not been reported to the best of our knowledge. There is a case report of a woman athlete with comorbid OCD and anorexia nervosa presenting with starvation resulting from compulsive exercising and restricted food intake, but she did not have any religious obsessions/compulsions.^3^

This report describes the case of an educated young man, who starved himself due to religious obsessions and compulsions (scrupulosity).

## THE CASE

A 25-year-old, postgraduate married Hindu man, presented with a history of 4 years of gradual change in behaviour in the form of repeated checking rituals, overzealous indulgence in religious activities, repeated thoughts that he might say or do something blasphemous. During the course of initial 2 years, he was able to perform his daily routine except for some difficulty in studies and no definitive treatment was sought. Gradually, his symptoms worsened, and his father brought him to psychiatry OPD for consultation. He was put on anti-obsessional treatment, but his compliance remained irregular and his condition fluctuating.

From about 3 months before the admission, his condition deteriorated further, he was noticed to get up early in the morning and become busy in stereotyped compulsive acts such as taking thorough bath with cold water (attributing religious significance to it) even in extreme winter season taking long time, purifying whole house including cowshed by worshiping and doing a lot of rituals such as sprinkling of water of river *Ganga* (considered holy water by Indians) in all rooms while reciting mantras, performing activities resembling *havan* (lighting sacred fire and putting herbs of religious significance in it) in cowshed in a stereotyped manner. He would spend hours together in these activities. Additionally, he used to wash utensils used for worshiping time and again, touch his parents' feet repeatedly and wash his hands and feet with ash so often that he developed skin lesions. During this period the patient also started fasting in religious context twice a week initially, which he gradually increased in frequency despite opposition from family members and from 1 month before admission he restricted his oral intake to a glass of milk and a banana once a day in the evening. From about 3 days before admission he stopped oral intake altogether. Patient became emaciated and quite weak to even walk and had to be brought to emergency department. He was then admitted to the Psychiatry ward.

On admission, the general physical examination revealed marked emaciation, cachexia and dehydration with prominent skin lesions on both palms and feet (due to repeated washing of hands). His weight was just 34 kg (50% less than his earlier weight) and blood pressure 90/70. On mental state examination, the patient was found to have irritable affect, repetitive and irrational ideas that he might annoy God by not performing ritualized religious acts with compulsion to perform the same. There was minimal resistance to stop these thoughts and activities despite considering them as absurd. Routine blood investigations were normal except haemoglobin 9 g%, fasting blood sugar 53 mg%.

The patient was maintained on i.v. fluids initially along with fluoxetine liquid 20 mg/day and risperidone liquid 2 mg/day in a disguised form as patient considered taking medications would break his fast. Over a period of one week fluoxetine was increased to 60 mg/day. After repeated sessions of psycho-education and coercion, he started accepting medica-tion orally after 2 days and food after 5 days of admission. After a week of admission, supportive psychotherapy and behaviour therapy (response prevention) were started. After about 3 weeks of regular combined pharmacotherapy, behaviour therapy and supportive psychotherapy, the patient gained a weight of 5 kg and showed significant improvement in his obsessive and compulsive symptoms and was discharged. He was still maintaining improvement after 6 months of discharge with body weight of 56 kg on last follow-up visit.

## DISCUSSION

OCD is one anxiety disorder that is potentially disabling condition that can persist throughout life. Those who suffer from OCD get trapped into a pattern of repetitive thoughts and behaviours, which they know are senseless or exaggerated but this knowledge remains insufficient to stop them obsessing or carrying out the rituals. Our patient fulfills the diagnostic criteria of OCD according to DSM-IV^4^ as he considered his thoughts and repetitive rituals as absurd and tried to resist them in the initial phase of disorder. However, later he had poor insight and there was minimal or no resistance. This is not an uncommon feature seen in course of OCD. In this case the patient was trapped into a pattern of repetitive rituals which led him to emaciation. Ritualistic eating patterns and weight loss can also be seen in patients with anorexia nervosa and anorexia can be a manifestation of OCD.^5^,^6^ But in this case there was no body image disturbance, an important feature seen in patients with anorexia nervosa.^4^ Additionally, this patient had very prominent obsessions and compulsion and decreased food intake was secondary to his religious obsessions and compulsions, so possibility of anorexia nervosa is unlikely in this case. His behaviour cannot be considered as features of psychotic disorder as the rituals were performed by him to relieve his anxiety which arose as a result of obsessive thoughts and he had full insight into his behavior and tried to resist them in the initial course of illness. Development of poor insight and minimal resistance to obsessions at a later stage may partially be attributed to intertwining of the obsessions and compulsions with his religious life.

It may be concluded that OCD may present with severe weight loss and it needs to be differentiated from other psychiatric illnesses associated with marked emaciation including anorexia nervosa.
